# Supplementary material for: Dual Higgs modes entangled into a soliton lattice in CuTe
Source: Nat Commun. 2024 Feb 2;15:984. doi: 10.1038/s41467-024-45354-4 (PMC10834594; doi:10.1038/s41467-024-45354-4)
Supplement: Supplementary file 1 — Supplementary information [file 41467_2024_45354_MOESM1_ESM.pdf]

Supplementary Information

SeongJin Kwon[1,2], Hyunjin Jung[1,2], SangJin Lee[1,2], Gil Young Cho[1,2], KiJeong Kong[1], ChoongJae Won[1,3,4], Sang-Wook Cheong[3,4,5], Han Woong Yeom[1,2†]

[1]*Center for Artificial Low Dimensional Electronic Systems, Institute for Basic Science, Pohang 37673, Korea.*

[2]*Department of Physics, Pohang University of Science and Technology, Pohang 37673, Korea.*

[3]*Laboatory for Pohang Emergent Materials, POSTECH, Pohang 37673, Korea*

[4]*MPPC-CPM, Max Planck POSTECH/Korea Research Initiative, Pohang 37673, Korea*

[5]*Rutgers Center for Emergent Materials and Department of Physics and Astronomy, Piscataway, New Jersey 08854, United States*

[†]*yeom@postech.ac.kr*

Charge-density-wave energy gap opening observed by angle-resolved photoelectron spectroscopy

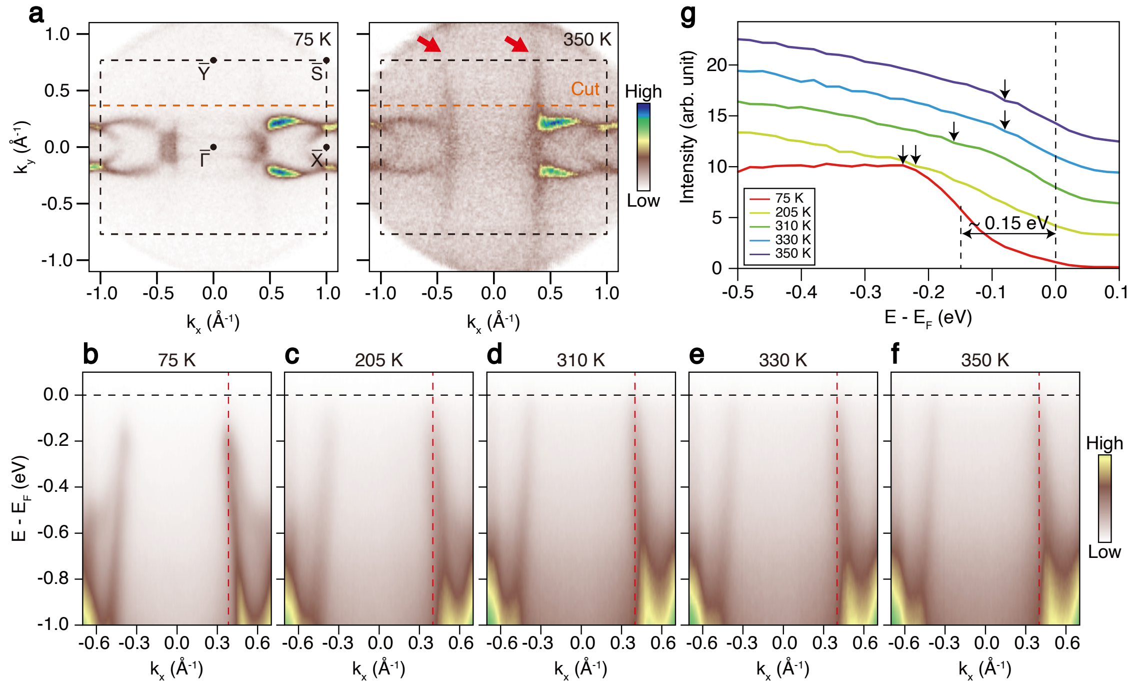

Supplementary Figure 1: a. Fermi contour map (the spectral intensity map at Fermi energy) at 75K and 350K, respectively, taken by angle-resolved photoemission spectroscopy (ARPES). Black dashed line shows the first Brillouin zone. b-f. Temperature evolution of electronic band dispersion for k_x at $k_y = 0.37\text{Å}^{-1}$ (along the orange dashed line in a). g. Temperature-dependent photoelectron energy distribution curves at $k_x = 0.4\text{Å}^{-1}$ (indicated with red lines in b-f) where the CDW energy gap develops on the 1D band at low temperature. Black arrows indicate the leading edges and the fully developed band gap has a size of 0.15eV below the Fermi energy.

Details on free energy and numerical simulations. Here we discuss some details of the free energy equation (Eq.1 in the main text) and the numerical simulations. The free energy density is

$$f = f_0 + f_{\text{int}}, \tag{1}$$

$$f_0 = \sum_{i=\alpha,\beta} \left(\frac{K}{2} \left| \frac{\partial \phi_i}{\partial x} \right|^2 + \frac{a}{2}|\phi_i|^2 + \frac{b}{5}(\phi_i^5 + \phi_i^{*5}) + \frac{c}{4}|\phi_i|^4 + \frac{d}{6}|\phi_i|^6 \right), \tag{2}$$

$$f_{\text{int}} = g_1 \frac{\partial \phi_\alpha^*}{\partial x}\frac{\partial \phi_\beta}{\partial x} + g_2 \phi_\alpha^* \phi_\beta + c.c + g_4 |\phi_\alpha|^2 |\phi_\beta|^2. \tag{3}$$

For simplicity, we will set $K = 1$ without losing generality. At $T < T_{CDW}$, we set $a < 0$. We tune g_2 and g_4 appropriately so that the two degenerate ground states are either $\langle \phi_\alpha \rangle = 0, \langle \phi_\beta \rangle \neq 0$, or $\langle \phi_\alpha \rangle \neq 0, \langle \phi_\beta \rangle = 0$. That is, the ground state is either exclusively the α CDW state or the β CDW state, but is never a mixture of both. We can now derive the equation of motions for the fields

$$\frac{\partial}{\partial x}\left(\frac{\delta f}{\delta\left(\partial A_i/\partial x\right)} \right) - \frac{\delta f}{\delta A_i} = 0, \;\; \frac{\partial}{\partial x}\left(\frac{\delta f}{\delta\left(\partial \theta_i/\partial x\right)} \right) - \frac{\delta f}{\delta \theta_i} = 0 \tag{4}$$

where we used $\phi_{i=\alpha,\beta} = A_i e^{i\theta_i}$. Here $A_i \in \mathbb{R}_+$ represents the amplitude and θ_i is the phase of the CDW order. We also impose $A_\alpha + A_\beta = A_0$, which is motivated by the experimental data (Fig. 3b and Supplementary Figure 10). For the stability of the oscillating solution, we impose $A_0 \ll |\langle \phi_0 \rangle|$ in which $\langle \phi_0 \rangle$ is the value of ϕ_β, which minimizes f_0 with $\phi_\alpha = 0$. This condition is needed for the curvature of the path of the order parameters $(\phi_\alpha, \phi_\beta)$ in the free energy landscape to be negative (Figs. 4a, 4b). Note that the negativity of the curvature for the path in the free energy landscape is also necessary for the soliton lattice solution for the ϕ^4-theory[1–3]. With these in mind, we next impose the boundary conditions as described in the main text to seek oscillating solutions. Unfortunately, unlike the case of a $(1+1)$D single (real) scalar ϕ^4-theory[1–3], an analytic solution for general symmetry groups and multiple components is not known in the literature. Hence, for

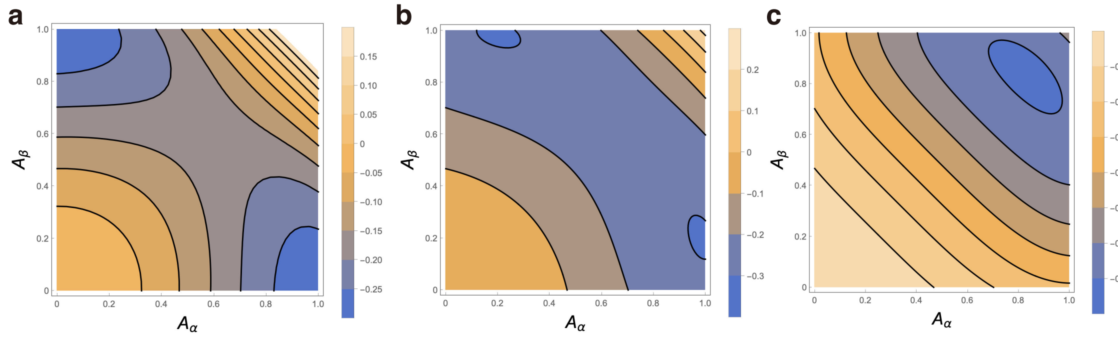

Supplementary Figure 2: Potential landscape with different $g_2(T)$ and fixed $g_4(T)$. We plot free energy potential, f_0 with $g_2(T) = a(T)$ (left), $g_2(T) = \left(-\frac{1}{5}\right) a(T)$ (center), $g_2(T) = (-1)a(T)$ (right) with fixed $g_4(T) = c(T) = a^{3/2}(T)$. The landscape on the leftmost figure has ground states: $\langle\phi_\alpha\rangle = 0$, $\langle\phi_\beta\rangle \neq 0$ and $\langle\phi_\alpha\rangle \neq 0$, $\langle\phi_\beta\rangle = 0$

our particular case, we have to resort to the numerical approach.

We next present our numerical solution, which is largely consistent with the experimentally observed behaviors. Here, we set $g_1 = 0$, $g_2 = 0.02a$ and $A_0 = 0.4|\langle\phi_0\rangle|$ on supplementary Figure 2. As mentioned in the main text, the amplitude oscillations can be well understood in a similar manner to those of ϕ^4-theory[1–3]. In this view, the condition $A_0 < |\langle\phi_0\rangle|$ basically confines the path of $(\langle\phi_\alpha\rangle, \langle\phi_\beta\rangle)$ in the free energy landscape to have a negative curvature (Figs. 4a, 4b).

The behaviors of $\theta_{\alpha,\beta}$ are also straightforward to understand. For clarity, let us consider the background where the amplitudes of the two CDWs oscillate in space. We will focus on how each term affects the evolution of the phase variables. First of all, the term $\propto b$ in Supplementary Equation 2 tends to pin down the phase variables to $\theta_{\alpha,\beta} = \frac{(2n+1)\pi}{5}$ with $n \in \mathbb{Z}$ for $b > 0$. Because of this term, the phases of the CDW orders typically form plateaus in space. However, the overall

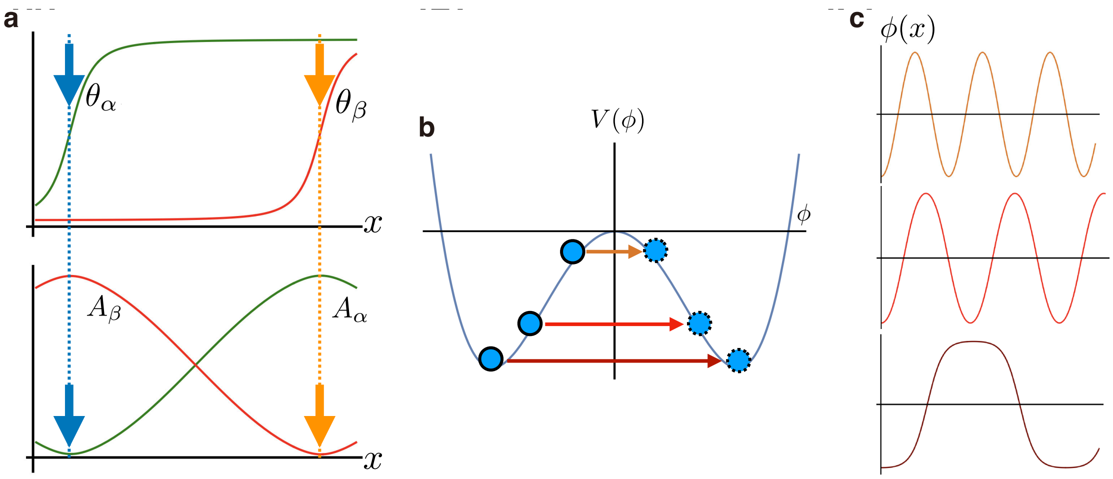

Supplementary Figure 3: a. Amplitudes and phases of the CDW order parameters. The top panel is the spatial profile of the phases, and the bottom one plots the amplitude of order parameters. Blue arrows represent the minima of A_α. Here, the corresponding phase variable θ_α slips by $+2\pi/5$, while θ_β is almost constant. Similarly, the orange arrow represents the position where the β CDW order parameter vanishes. b,c. A potential of ϕ^4-theory and corresponding soliton lattice solutions with various amplitudes (color coded). The size of oscillating amplitudes in Supplementary Figure 3b gives a real-space profile of soliton lattice solutions labeled by the same colors in Supplementary Figure 3c. Note that periods of the soliton lattice in Supplementary Figure 3c is roughly proportional to the oscillating amplitudes in Supplementary Figure 3b.

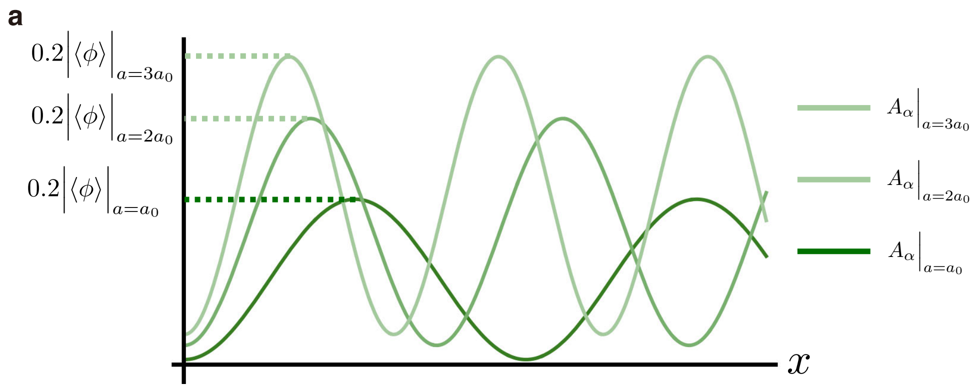

Supplementary Figure 4: Periods of amplitude oscillation of $A_\alpha(x)$ as a function of $a(T)$. We observe that the periods and amplitudes decrease as increasing $a(T)$. Intuitively, as we increase $a(T)$, the potential becomes steeper, which will make the fields oscillate faster in space. This is consistent with what one would expect from the lessons of the ϕ^4 theory (See Supplementary Figures 5b and 5c.).

strength of this term is proportional to the amplitudes. Hence, when the amplitude of one CDW order vanishes, the corresponding phase variable can slip in space and form the domain walls, e.g., $\theta_\alpha \to \theta_\alpha \pm 2\pi/5$ across the point (blue arrow at Supplementary Figure 3a) when $A_\alpha \to 0$. At this moment, the amplitude of the other CDW, i.e., $A_\beta \neq 0$, is however robust and so θ_β is also well pinned for this region. However, there is another term $\propto g_2$ (where we set g_2 positive), which tries to make $\theta_\alpha = \theta_\beta$ in space. Hence, when the amplitude of $A_\beta \to 0$ while $A_\alpha \neq 0$ (orange arrow at Supplementary Figure 3a), this term induces θ_β to change in the manner that θ_β follows θ_α. Hence, we see the intertwined oscillations of the phases and amplitudes.

Numerically we find that the period of the oscillation depends on the parameters of our theory. For example, if we tune $a(T) \propto (T - T_{CDW})$, then we can see the change in the periods

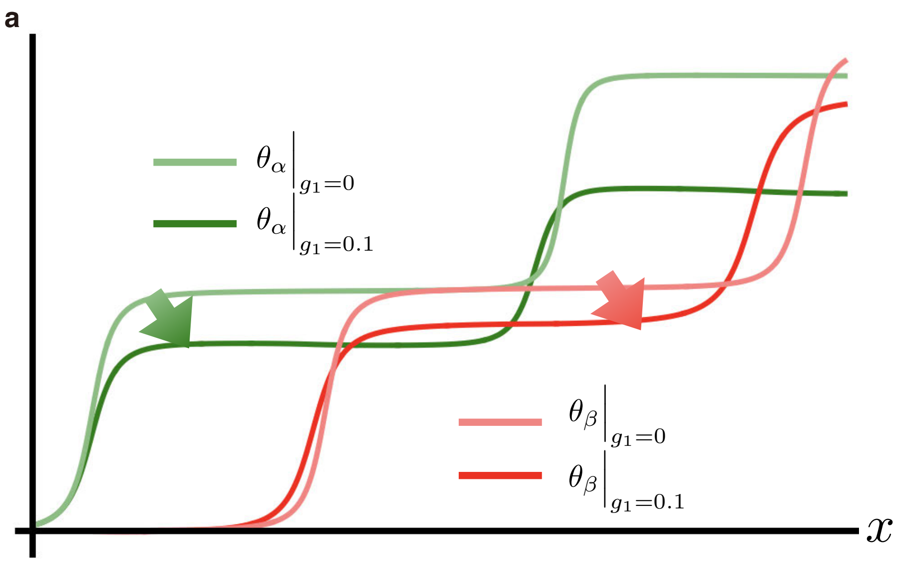

Supplementary Figure 5: Finite g_1 effects. Turning on g_1 slightly tilts and shifts the position of the plateau in the $g_1 = 0$ limit. Finite $g_1 \frac{\partial \phi_\alpha^*}{\partial x} \frac{\partial \phi_\beta}{\partial x} + c.c.$ prefers tilted plateaus and additionally gained free energy shifts the position of plateaus.

of the oscillation of the amplitude $A_\alpha(x)$ (See Supplementary Figure 4). Here, we set a_0 as a reference scale that we used in Supplementary Figure 4, and we tune a/a_0. As we increase a/a_0, the period decreases because of the larger curvature of the potential. This is actually what one expects from the analogy with the ϕ^4-theory, where the period of the oscillation is shorter for the steeper potentials. Moreover, its period also depends on the combination of coefficients of quadratic and quartic terms and the amplitude (See Supplementary Figures 3b and 3c.).[1–3].

Finally, let us comment on the effect of g_1. This term tends to round the plateaus. We numerically solve the equation of motions with $g_1 = 0.1K$ (See Supplementary Figure 5). We observe that the originally flat plateaus were tilted and shifted from the $g_1 = 0$ case which can be

understood as follows. The derivative couplings prefer small tilting in the original plateau regime of θ to reduce free energy and these can shift the position of plateaus.

Details of the scanning tunneling microscopy topography

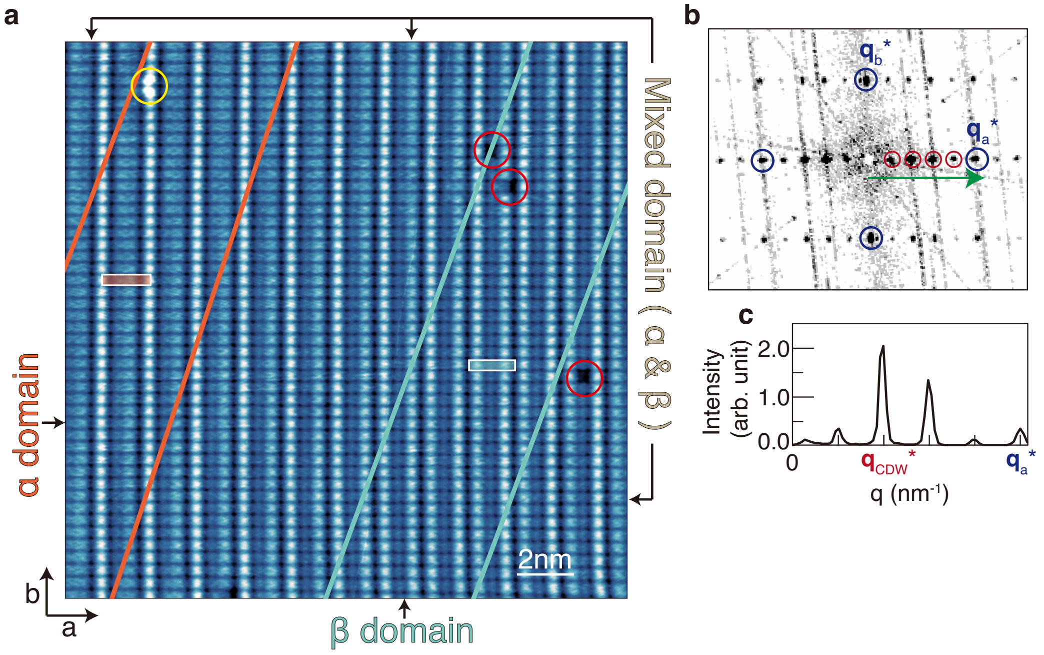

Supplementary Figure 6: a. Atom-resolved STM topography of CuTe at a sample bias of 500 mV. Yellow and red circles indicate the defects. The lateral heterostructures between α and β CDW states are observed. b,c. 2D FFT image of the STM topograph of a and its intensity profile along q_a* (the green arrow in b), which shows a clear CDW modulation of $5a_0$ periodicity.

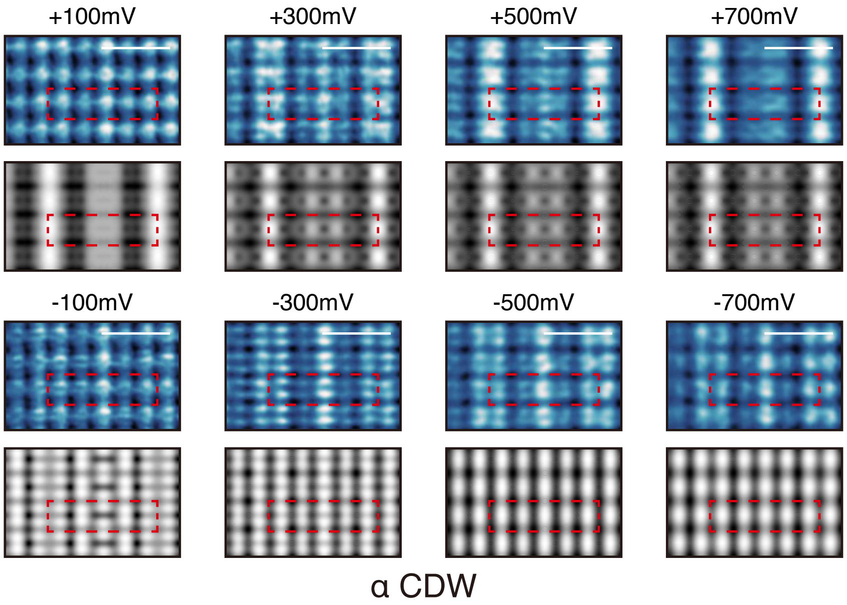

Supplementary Figure 7: Bias dependent STM topographies and corresponding DFT-simulated images of the α CDW state. Red dotted boxes indicate the 5×1 CDW unitcell. The white scale bar represents 1nm length.

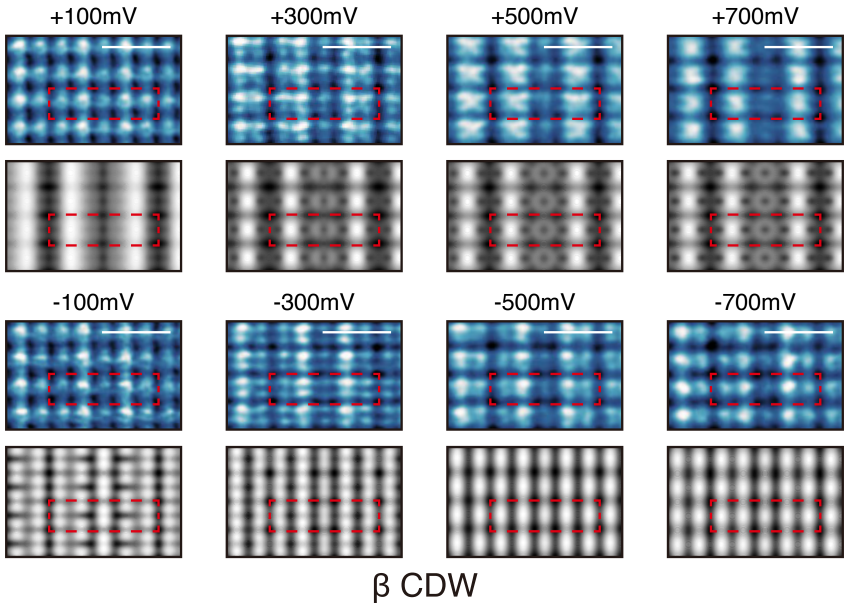

Supplementary Figure 8: Bias dependent STM topographies and corresponding DFT-simulated images of the β CDW state. Red dotted boxes indicate the 5×1 CDW unitcell. The white scale bar represents 1nm length.

Details of lateral CDW amplitude variations

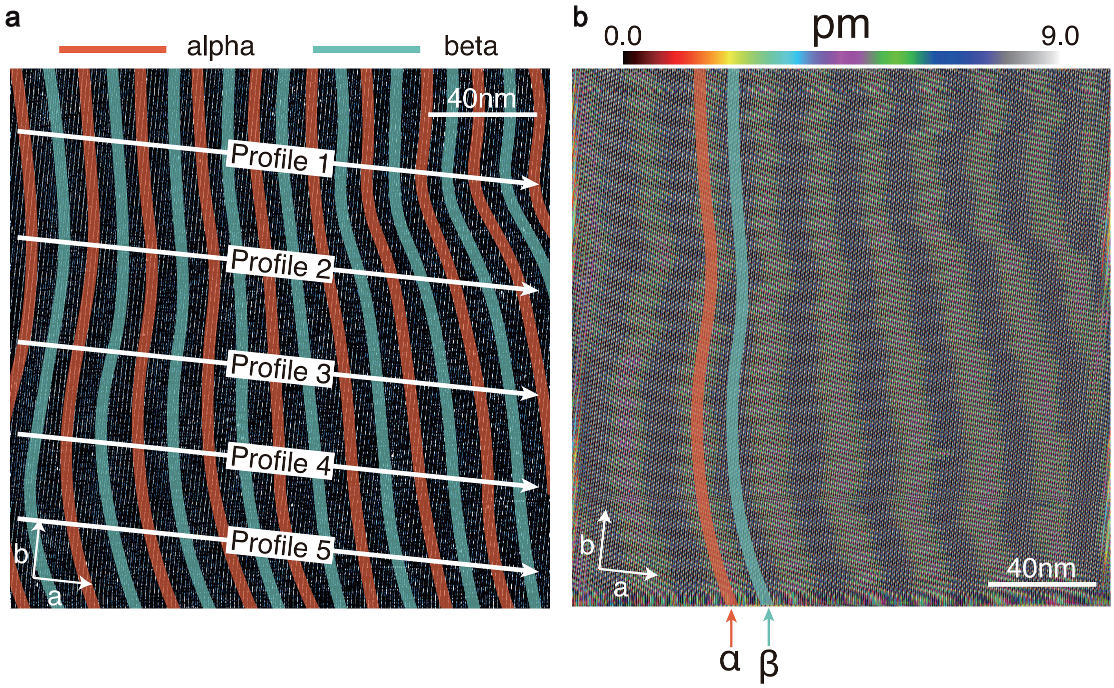

Supplementary Figure 9: a. Wide range (200×200 nm) STM topography with the CDW domains colored at bias voltage 700mV. The colored domain configuration represents a smectic (stripe) order of degenerate CDWs. b. Same topography with different color scale to emphasize the stripe pattern. (See Suppelmentary Fig. 10.).

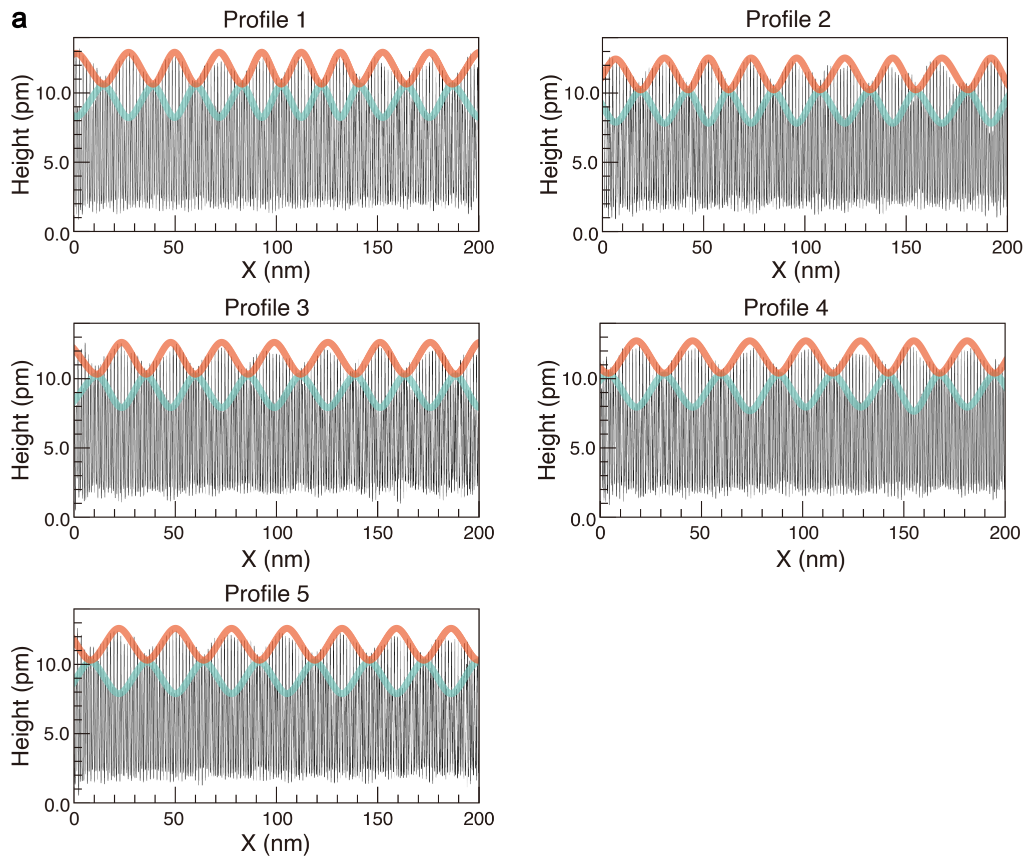

Supplementary Figure 10: a. Examples of height profiles extracted along the white arrows in Supplementary Figure 9. The clear oscillatory patterns along a-axis are observed. The amplitude oscillations of the two main topography peak are fitted well by Jacobi elliptic sine function, which is the general solution of one-dimensional soliton lattice as overlaid in red and blue lines. The initial free parameter of the function is fitted with -0.4 and amplitude waves have a wavelength of 24 nm in average with the standard deviation of 2.9 nm. The height difference between α and β states remains almost same over the whole surface area measured.

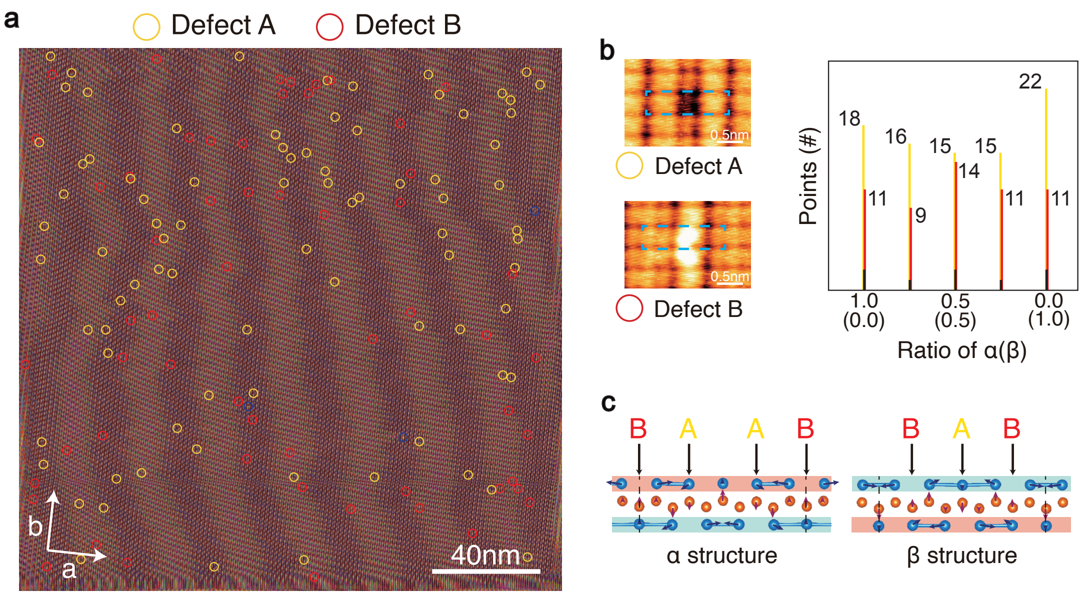

Supplementary Figure 11: a. Large-scale STM images at bias voltage 700 mV showing the positions of two major types of defects (A and B defects in yellow and red circles, respectively) and the amplitude modes (α and β phases in bright and dark contrast, respectively). b. Enlarged STM images at bias voltage 500 mV of A and B defects with their relative populations within a half period of the amplitude wave [from the maximum of the $\alpha(\beta)$ phase to the neighboring minimum of it]. Type A defects favor being located at the maximum or minimum of α (or β) phase while type B defects have a weaker tendency to be located in the transition region between the two phases. c. Atomic scale location of the A and B type defects within the α and β structures. The atomic structures of the defects are not decided at present. 5×1 unitcell is indicated as dotted lines.

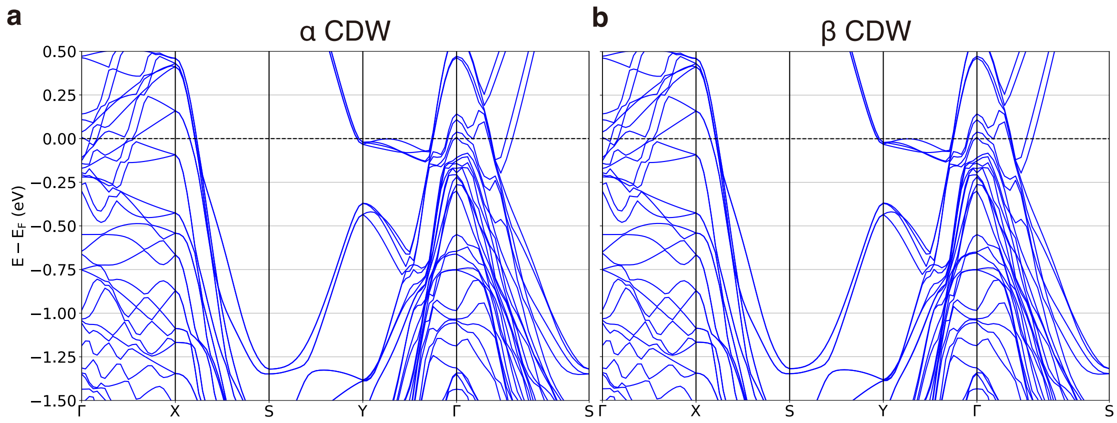

Supplementary Figure 12: a. Calculated band structures of α (left) and β (right) CDW states. Two band structures are identical as represented by the CDW gap at Y point. The total energy difference between the two is within the numerical error, which confirms the degeneracy of the two CDW states.

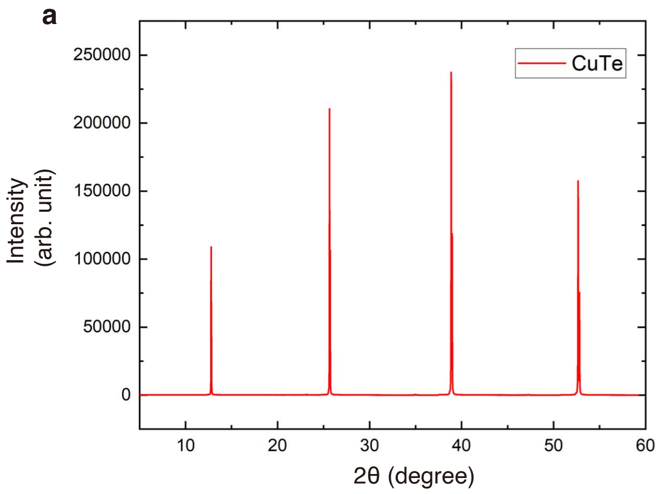

Supplementary Figure 13: Cu Kα X-ray diffraction spectrum of CuTe single crystals at room temperature. All sharp peaks can be attributed to the (00l) planes of CuTe, ranging from (001) to (004).

1. Carrillo, J. E., Maia Jr, A. & Mostepanenko, V. JACOBI ELLIPTIC SOLUTIONS OF $\lambda\phi^4$ THEORY IN A FINITE DOMAIN. *International Journal of Modern Physics A* **15**, 2645–2659 (2000).

2. Lizunova, M. & van Wezel, J. An introduction to kinks in φ^4-theory. *SciPost Phys. Lect. Notes* 23 (2021).

3. Abramowitz, M. & Stegun, I. A. *Handbook of mathematical functions with formulas, graphs, and mathematical tables*, vol. 55 (US Government printing office, 1968).
